# Targeting the cyclin D/CDK4 Axis to unlock new therapeutic approaches to enhance cancer treatment

**DOI:** 10.1371/journal.pone.0330102

**Published:** 2025-08-22

**Authors:** Ibrahim Khater, Aaya Nassar

**Affiliations:** Biophysics Department, Faculty of Science, Cairo University, Giza, Egypt; University of Mashreq, IRAQ

## Abstract

Dysregulation of the cyclin D/CDK complex is a common feature in various cancers, including colorectal, breast, and melanoma, leading to uncontrolled tumor growth and cell cycle progression. Targeting this complex has become a compelling therapeutic approach in oncology. FDA-approved CDK4/6 inhibitors, such as ribociclib, palbociclib, and abemaciclib, have demonstrated clinical efficacy, significantly improving patient outcomes. However, the development of resistance to these therapies emphasizes the urgent need for alternative strategies to overcome therapeutic limitations. The current study explores the potential of new inhibitors targeting the cyclin D/CDK axis by using virtual screening and molecular dynamics simulations. We conducted a virtual screening of the Zinc and PubChem databases, by utilizing a pharmacophore model generated by PocketQuery, to identify new candidate inhibitors of the cyclin D/CDK4 complex. The findings offer promising leads for further optimization, potentially paving the way for developing more effective treatments that circumvent resistance mechanisms and expand therapeutic options for cancer patients. Further experimental validation and in vivo studies are necessary to confirm the efficacy of these candidates and translate them into viable clinical treatments.

## 1. Introduction

The cell cycle is a tightly regulated process that ensures the accurate replication and division of cells, progressing through distinct phases: G1 (first gap), S (synthesis), G2 (second gap), and M (mitosis). Each phase transition is controlled by specific proteins and complexes that act as checkpoints, preserving cellular integrity and preventing abnormal proliferation. A central regulator in this process is the cyclin D–CDK4/6 complex, which drives the G1 phase by phosphorylating and inactivating the retinoblastoma protein (pRb), a tumor suppressor that binds to and inhibits E2F transcription factors essential for G1 to S phase progression [1–4]. When pRb is phosphorylated, it releases E2F, triggering the transcription of genes required for DNA replication, thereby facilitating controlled cell growth and division.

Growth factor signaling pathways activate the cyclin D–CDK4/6 complex, leading to elevated cyclin D expression, which binds to and activates CDK4/6. This activation results in pRb phosphorylation, E2F release, and cell cycle progression [5,6]. Dysregulation of the cyclin D–CDK4/6 complex is linked to various cancers, where increased cyclin D production drives the activation of CDK4/6 and abnormal cell cycle advancement [7]. Consequently, inhibiting the cyclin D–CDK4/6 complex has emerged as a promising therapeutic approach in cancer management. CDK4/6 inhibitors, such as palbociclib, ribociclib, and abemaciclib, are used clinically for cancers with cyclin D amplification, including breast cancer [8,9]. These inhibitors function by binding to the ATP-binding pocket of CDK4/6, impeding kinase activity and arresting cell proliferation [10].

However, resistance to CDK4/6 inhibitors, driven by alternative pathway activation, CDK4/6 gene mutations, or pRb pathway alterations, presents a significant challenge [11]. As a result, targeting the broader cyclin/CDK axis rather than CDKs alone offers a promising strategy with potential benefits [10,12,13]. This axis encompasses various cyclin-CDK interactions, each regulating distinct stages of the cell cycle. By focusing on specific cyclin/CDK complexes linked to cancer, targeting this axis could minimize toxicity and enhance selectivity, as inhibiting CDK activity alone can impact additional CDK roles beyond the cell cycle, leading to side effects [10,13–16]. Moreover, targeting the cyclin/CDK axis may address CDK inhibitor resistance and provide more precise, individualized therapeutic interventions.

Given the role of the cyclin/CDK axis in cell cycle regulation and the aberrant activation of this pathway in numerous cancers, targeting this axis may open novel therapeutic paths.

## 2. Methods

### 2.1. Selection and preparation of proteins

The crystal structure of CDK4 in complex with a D-type cyclin (PDB ID: 2W99) was obtained from the Protein Data Bank (https://www.rcsb.org). To prepare the structure for analysis, heteroatoms and water molecules were removed using PyMol software to ensure focus on the primary protein-protein interaction site [17].

For identifying and designing a pharmacophore targeting the CDK4-cyclin D interaction, PocketQuery was utilized (http://pocketquery.csb.pitt.edu), a tool developed by David Koes at the University of Pittsburgh’s Department of Computational and Systems Biology in the Camacho Lab. PocketQuery enables users to visualize “hot spots” at the protein-protein interface, identifying key clusters and anchor residues that contribute to binding specificity.

The pharmacophore model generated by PocketQuery was then exported to ZincPharmer (http://zincpharmer.csb.pitt.edu/pharmer.html) to search for structurally similar ligands within the Zinc database, optimizing for potential compounds that could effectively target the interaction site [18]. This approach enables a focused selection of candidates for further molecular dynamics simulations and virtual screening to assess binding interactions with the CDK4-cyclin D complex.

### 2.2. Ligand optimization and molecular docking

Ligands were optimized using the MMFF94 force field in Avogadro software [19,20] (http://avogadro.cc) to achieve stable conformations prior to docking. Molecular docking was performed using AutoDock Vina (http://vina.scripps.edu) [21] under default parameters. Polar hydrogens were added to the amino acid residues of the protein structure, followed by the application of Kollman charges [22].

Docking was guided by non-covalent protein-ligand interactions, including hydrogen bonds, Coulomb interactions, Van der Waals forces, and electrostatic interactions, to evaluate potential binding sites and determine binding affinities. Automation of various tasks, such as generation of multiple ligand ‘pdbqt’ files and receptor configurations, was efficiently handled by the Raccoon interface [23]. The ligands were treated as flexible during docking, while the protein remained rigid.

Using the AutoDock 4 platform’s AutoDock and AutoGrid tools, we generated the necessary docking and grid parameter files [24]. Binding sites were ranked based on docking scores, which were computed as the Gibbs free energy of binding (ΔG) or inhibition constant (Ki) values. Each ligand underwent five docking runs to obtain consistent scores, after which the average and standard deviation were calculated. Final docking results were saved in PDB format for further analysis [21].

### 2.3. Pharmacokinetic (ADME)

Pharmacokinetic properties, including blood-brain barrier (BBB) penetration, human intestinal absorption percentage, and plasma protein binding, were predicted using the online SwissADME service (http://swissadme.ch/index.php) [25]. SwissADME calculates these properties for bioactive compounds to assess their potential for drug-like behavior. Additionally, the toxicity of the chemicals was evaluated using ProTox 3.0 (https://tox.charite.de/protox3), a tool for predicting the toxicity of various substances.

### 2.4. Interactions between ligands and proteins

The interactions between the cyclin D/CDK4 complex and ligands were analyzed using the Protein-Ligand Interaction Profiler (PLIP) web server (https://plip-tool.biotec.tu-dresden.de/plip-web/plip/index). This tool provides detailed insights into various types of protein-ligand interactions, including hydrophobic interactions, hydrogen bonds, p-cation interactions, and halogen bonds. The interaction patterns were identified and visualized from 3D structures using PLIP. Subsequently, the results were further analyzed and presented as both 2D and 3D interaction diagrams using PyMol and Discovery Studio visualizer software, allowing a comprehensive examination of each binding site.

### 2.5. Molecular dynamics simulation

The molecular dynamics simulation (MDS) was conducted using the CHARMM36 force field and the GROMACS-2019 software suite [26,27]. Protein topology and ligand parameter files were generated using CHARMM-GUI. Water molecules were replaced by Cl- ions, and the protein-ligand complex was solvated and neutralized. Following these preparations, the system was equilibrated for 125 ps under constant number of molecules, volume, and temperature (NVT) conditions. After equilibration, the system underwent energy minimization for 5000 steps using the gradient descent method. The final molecular dynamics simulation was performed for 100 ns with a time step of 2 fs, maintaining constant pressure and temperature (310 K). During the simulation, key structural properties such as the Radius of Gyration (Rg), Root Mean Square Deviation (RMSD), and Root Mean Square Fluctuation (RMSF) were calculated to analyze the stability and flexibility of the protein-ligand complex.

## 3. Results and discussion

### 3.1. Pharmacophore extraction

**Fig 1:**
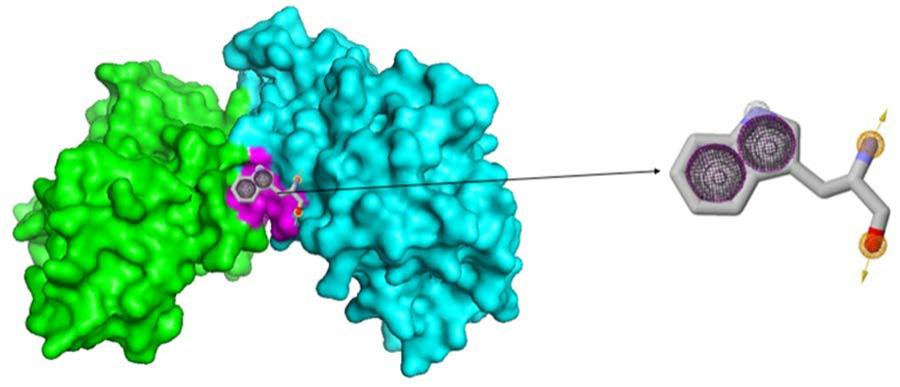
Crystal structure of the 2W99 complex, displaying chain A (cyclin D) in green and chain B (CDK4) in cyan. The functionally significant binding pocket, highlighted in magenta, includes the pharmacophore designed by PocketQuery.

A pharmacophore was designed using PocketQuery (http://pocketquery.csb.pitt.edu), and the protein-protein interactions within the crystal structure of the cyclin D/CDK4 complex (PDB ID: 2W99) were analyzed (Table 1, Fig 1). To identify ligands similar to the created pharmacophore, it was exported to Zincpharmer (http://zincpharmer.csb.pitt.edu), a database for virtual screening. The search returned 40 ligands with the lowest mean square difference to the pharmacophore, which were selected for further evaluation.

**Table 1:** Features of the pharmacophore designed using PocketQuery.

Pharmacophore Class	x	y	z	Radius
Hydrophobic	11.21	20.86	27.10	1.00
Hydrophobic	10.56	22.90	26.89	1.00
Aromatic	11.21	20.86	27.10	1.10
Aromatic	10.56	22.90	26.89	1.10
Hydrogen Donor	11.13	26.45	28.31	0.50
Hydrogen Donor	9.44	22.85	26.54	0.50
Hydrogen Acceptor	11.13	26.45	28.31	0.50
Hydrogen Acceptor	14.60	25.76	29.00	0.50

### 3.2. Molecular docking

Approximately forty ligands obtained from ZincPharmer were docked into the solved structure of the cyclin D/CDK4 complex (PDB ID: 2W99) using AutoDock Vina software. The docking process involved optimizing the ligands for non-covalent interactions, incorporating polar hydrogen atoms, applying Kollman charges to the protein, and performing geometry optimization and energy minimization. The docking scores and their associated standard deviations are summarized in Table 2. Among the ligands, Z33 achieved a docking score of −9.7 kcal/mol.

**Table 2:** Docking scores (kcal/mol) for the 2W99 complex were determined using AutoDock Vina.

Compound	Zinc ID	Docking score	Standard Deviation	Compound	Zinc ID	Docking score	Standard Deviation
Z1	ZINC12401855	−6.4	0.30	Z21	ZINC16021426	−9.02	0.09
Z2	ZINC83301209	−8.6	0	Z22	ZINC13575614	−8.74	0.23
Z3	ZINC20757631	−7.56	0.20	Z23	ZINC15970445	−8.2	0.08
Z4	ZINC20756821	−8.1	0	Z24	ZINC15970075	−8.04	0.04
Z5	ZINC20567209	−8.18	0.14	Z25	ZINC15970359	−8.06	0.26
Z6	ZINC20611016	−8.76	0.1	Z26	ZINC16020253	−9.3	0.2
Z7	ZINC16025055	−8.44	0.24	Z27	ZINC16020253	−9.4	0.22
Z8	ZINC20757468	−7.82	0.07	Z28	ZINC16021667	−9.14	0.08
Z9	ZINC20757502	−7.98	0.36	Z29	ZINC16024862	−8.62	0.04
Z10	ZINC20757382	−7.82	0.13	Z30	ZINC15970502	−7.86	0.19
Z11	ZINC20756905	−8.06	0.2	Z31	ZINC16022315	−8.1	0.12
Z12	ZINC20757233	−7.84	0.08	Z32	ZINC16025057	−8.78	0.24
Z13	ZINC16021666	−8.84	0.08	Z33	ZINC16021428	−9.7	0.2
Z14	ZINC20757008	−7.78	0.04	Z34	ZINC20757456	−7.86	0.08
Z15	ZINC20757172	−8.2	0	Z35	ZINC15957594	−9.12	0.19
Z16	ZINC13573775	−7.88	0.29	Z36	ZINC02539441	−6.72	0.04
Z17	ZINC20756982	−7.8	0.26	Z37	ZINC39777971	−9.4	0
Z18	ZINC13574358	−8.18	0.07	Z38	ZINC35807468	−8.7	0
Z19	ZINC13575082	−8.26	0.04	Z39	ZINC35806836	−9.02	0.09
Z20	ZINC16020251	−8.78	0.04	Z40	ZINC13557523	−8.74	0.23

To identify compounds similar to Z33, we searched the PubChem database and selected seven comparable chemicals for further analysis. Their corresponding docking scores are listed in Table 3, with compound C2 showing a docking score of −9.88 kcal/mol.

**Table 3:** Docking scores (kcal/mol) for 2W99 and compounds similar to Z33, determined using AutoDock Vina.

Similar Compounds to Z33	ID	Compound name	Docking score	Standard deviation
Z33	ZINC16021428	(3aS,4S,6R,6aS)-5’-chloro-2-cyclohexyl-4-(1H-indol-3-ylmethyl) spiro[3a,4,5,6a-tetrahydropyrrolo[3,4-c]	−9.7	0.2
C1	CID_4886020	5’-(2-fluorophenyl)-3’-(1H-indol-3-ylmethyl)-3a’,6a’-dihydro-2’H-spiro[indole-3,1’-pyrrolo[3,4-c]pyrrole]-2,4’,6’(1H,3’H,5’H)-trione	−9.16	0.048989795
C2	CID_4887687	1-(1H-indol-3-ylmethyl)-5-[3-(trifluoromethyl)phenyl]spiro[1,2,3a,6a-tetrahydropyrrolo[3,4-c]pyrrole-3,3’-1H-indole]-2’,4,6-trione	−9.88	0.074833148
C3	CID_4896022	5-butan-2-yl-5’-fluoro-1-(1H-indol-3-ylmethyl)spiro[1,2,3a,6a-tetrahydropyrrolo[3,4-c]pyrrole-3,3’-1H-indole]-2’,4,6-trione	−8.38	0.146969385
C4	CID_166441662	(3S,3aS,6aR)-3-[2-(1H-indol-5-yl)phenyl]-2-methyl-5-phenyl-1,3,3a,6a-tetrahydropyrrolo[3,4-c]pyrrole-4,6-dione	−8.38	0.116619038
C5	CID_72197302	1-[4-[4-(5-chloro-1H-indol-3-yl)-3,6-dihydro-2H-pyridin-1-yl]butyl]-3-(1H-indol-3-yl)pyrrolidine-2,5-dione	−8.04	0.392937654
C6	CID_72197477	1-[3-[4-(5-chloro-1H-indol-3-yl)-3,6-dihydro-2H-pyridin-1-yl]propyl]-3-(1H-indol-3-yl)pyrrolidine-2,5-dione	−8.06	0.1356466
C7	CID_72197665	1-[2-[4-(5-chloro-1H-indol-3-yl)-3,6-dihydro-2H-pyridin-1-yl]ethyl]-3-(1H-indol-3-yl)pyrrolidine-2,5-dione	−9.16	0.048989795

### 3.3. Pharmacokinetic (ADME) and *In Silico* Studies Profile

The physicochemical properties of the highly active derivatives were assessed, and an in-silico report was generated to predict their ADMET (Absorption, Distribution, Metabolism, Excretion, and Toxicity) profiles. The molecules were evaluated for their compliance with the “rule of five,” which predicts good absorption potential if at least three of the following criteria are met: the partition coefficient (log P) should not exceed 5, molecular weight (MW) should not exceed 500, the number of hydrogen bond donors (H-bond donors) should be no more than 5, and the number of hydrogen bond acceptors (H-bond acceptors) should be no more than 10 [28].

**Fig 2:**
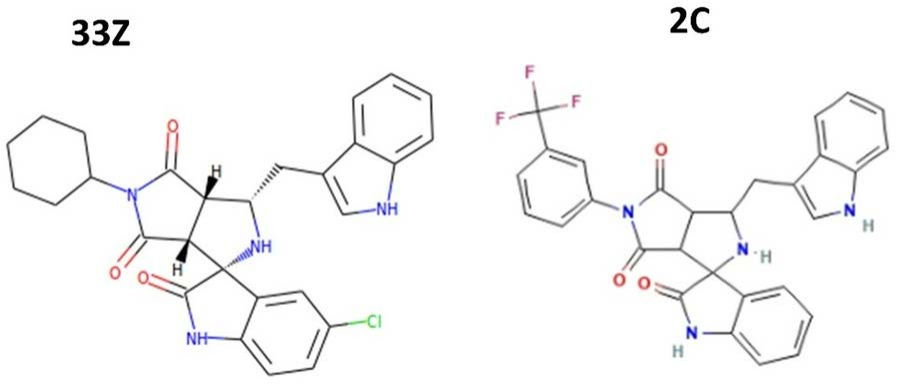
The Chemical structures of compounds Z33 and C2 were identified through virtual screening of the Zinc and PubChem databases.

Based on the results presented in Table 4, compounds Z33 and C2 (Fig 2) showed good gastrointestinal tract (GIT) absorption, suggesting they are likely to pass through biological membranes efficiently. However, compound Z33 exhibited two violations of the “rule of five,” its molecular weight (502.99 g/mol) exceeded 500, and it had four H-bond acceptors. Compound C2 showed one violation with a molecular weight of 530.5 g/mol and seven H-bond acceptors. Both compounds showed no ability to cross the blood-brain barrier (BBB).

**Table 4:** Pharmacokinetic (ADME) profile of the highly effective compound C2, generated using the SwissADME server.

Parameter		Z33	C2
Physicochemical properties	Formula	C28H27ClN4O3	C29H21F3N4O3
	Molecular weight	502.99 g/mol	530.50 g/mol
	Num. heavy atoms	36	39
	Num. aromatic heavy atoms	15	21
	Fraction Csp3	0.39	0.21
	Num. rotatable bonds	3	4
	Num. H-bond acceptors	4	7
	Num. H-bond donors	3	3
	Molar refractivity	148.24	146.55
	TPSA	94.30 Å²	94.30 Å²
Lipophilicity	Log Po/w (iLOGP)	3.39	2.99
	Log Po/w (XLOGP3)	3.65	3.65
	Log Po/w (WLOGP)	2.68	4.07
	Log Po/w (MLOGP)	3.22	3.56
	Log Po/w (SILICOS-IT)	4.22	4.55
	Consensus Log Po/w	3.43	3.76
Water solubility	Log S (ESOL)	−5.37	−5.56
	Solubility	4.28e-06 mol/l	2.73e-06 mol/l
	Class	Moderately soluble	Moderately soluble
	Log S (Ali)	−5.32	−5.32
	Solubility	4.80e-06 mol/l	4.80e-06 mol/l
	Class	Moderately soluble	Moderately soluble
	Log S (SILICOS-IT)	−8.41	−9.73
	Solubility	3.88e-09 mol/l	1.86e-10 mol/l
	Class	Poorly soluble	Poorly soluble
Pharmacokinetics	GI absorption	High	High
	BBB permeant	No	No
	P-gp substrate	Yes	Yes
	CYP1A2 inhibitor	No	Yes
	CYP2C19 inhibitor	Yes	No
	CYP2C9 inhibitor	Yes	Yes
	CYP2D6 inhibitor	Yes	Yes
	CYP3A4 inhibitor	Yes	No
	Log Kp (skin permeation)	−6.78 cm/s	−6.94 cm/s
Drug likeness	Lipinski	Yes; 1 violation: MW > 500	Yes; 1 violation: MW > 500
	Ghose	No; 2 violations: MW > 480, MR > 130	No; 2 violations: MW > 480, MR > 130
	Veber	Yes	Yes
	Egan	Yes	Yes
	Muegge	Yes	Yes
	Bioavailability Score	0.55	0.55

Regarding metabolic properties, compound C2 was predicted to inhibit CYP3A4, a key enzyme involved in drug metabolism, whereas Z33 was not expected to inhibit this enzyme. Both compounds showed similar skin penetration rates (−6.78 cm/s for Z33 and −6.94 cm/s for C2). For lipophilicity, Z33 had a favorable Consensus Log Po/w of 3.76, while C2 had a lower value of 3.43. Both compounds were moderately soluble in water, as indicated by their Log S (ESOL) and Log S (Ali) values, although they were poorly soluble in the SILICOS-IT model.

Synthetic accessibility was also assessed, with compound C2 having a better synthetic accessibility score (4.91) compared to Z33 (5.01).

Both compounds passed the Lipinski filter for drug-likeness (MW ≤ 500, MLOGP ≤ 4.15, N or O ≤ 10, NH or OH ≤ 5) with one violation [29]. They also passed the Veber [30], Egan [31], and Muegge [32] filters without violations. However, both failed to meet the Ghose [33] filter (160 ≤ MW ≤ 480, −0.4 ≤ WLOGP ≤ 5.6, 40 ≤ MR ≤ 130, 20 ≤ atoms ≤ 70) due to two violations: MW > 480 and MR > 130.

**Fig 3:**
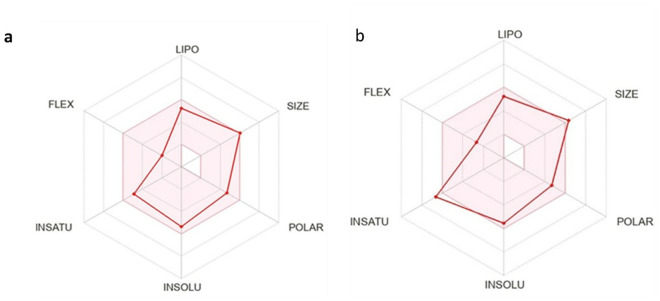
Bioavailability radar plots for the selected compounds, (a) Z33 and (b) C2, depicting their pharmacokinetic profiles.

The bioavailability of these compounds was evaluated using the bioavailability radar plot from the SwissADME server [25], as shown in Fig 3. The radar plots for both drugs indicate that they lie within the optimal physicochemical space for oral bioavailability, with a bioavailability score of 0.55 for both compounds.

Lastly, the toxicity of Z33 and C2 was predicted using the ProTox 3.0 server, which estimated their LD50 values to be 2000 mg/kg and 1190 mg/kg, respectively. Both compounds were classified in toxicity class 4, indicating that they could be harmful if consumed.

### 3.4. Analysis of Interactions between protein and ligand

**Fig 4:**
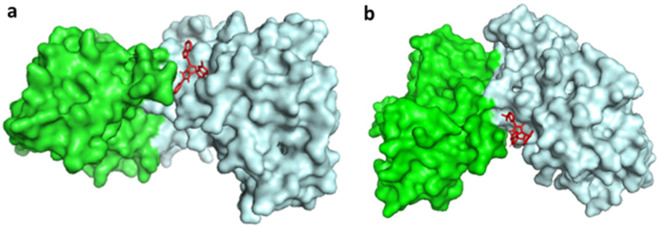
Binding sites of compounds (a) Z33 and (b) C2 within the active site cavity of the cyclin D/CDK4 complex, visualized through molecular docking studies.

The molecules Z33 and C2, which are docked to the 2W99 structure, exhibit docking scores of −9.7 and −9.88 kcal/mol, respectively. After the docking process, the 3D interactions between Z33 and 2W99, and C2 and 2W99, are shown in Fig 4 (a and b). The binding free energy for these interactions was calculated by summing the covalent energies from four key components: van der Waals forces, hydrogen bonds, desolvation energy, and electrostatic energy.

The Protein-Ligand Interaction Profiler (PLIP) server was used to analyze the interactions between Z33, C2, and 2W99. The main interactions are summarized in Table 5. For Z33, one hydrogen bond (H-bond) was identified between ARG126 of the D-type cyclin and the nitrogen atom of the pyrrole ring at a distance of 3.45 Å. In the case of C2, four H-bonds were formed with 2W99: one at a distance of 3.19 Å between ARG26 of CDK4, one at 3.71 Å between ARG126 of the D-type cyclin and the nitrogen atom of the pyrrole ring, and two at 3.07 Å involving ASP129 of the D-type cyclin, with one bond between the NH group and one between the oxygen atom.

**Table 5:** The interactions between Z33, C2, and 2W99 were analyzed by the Protein-Ligand Interaction Profiler (PLIP) server.

	Z33						C2					
**Hydrogen bond**	**Residue**	**AA**	**Distance H-A**	**Distance D-A**	**Donor Angle**	**Donor Atom**	**Residue**	**AA**	**Distance H-A**	**Distance D-A**	**Donor Angle**	**Donor Atom**
	126B	ARG	2.95	3.45	112.98	4 [Nam]	26A	ARG	2.72	3.19	109.99	43 [Ng+]
							126B	ARG	3.19	3.71	113.71	4263 [Npl]
							129B	ASP	2.77	3.07	100.09	2943 [O3]
							129B	ASP	2.13	3.07	153.03	4261 [N3]
**π-Stacking**							**Residue**	**AA**	**Distance**	**Angle**	**Residue**	**Ligand Atoms**
							68B	HIS	3.85	20.19	68B	4265, 4266, 4267, 4268, 4269, 4270

Additionally, a π-stacking interaction was observed between the phenyl ring of 3-(trifluoromethyl) phenyl and HIS68 of the D-type cyclin at a distance of 3.85 Å.

**Fig 5:**
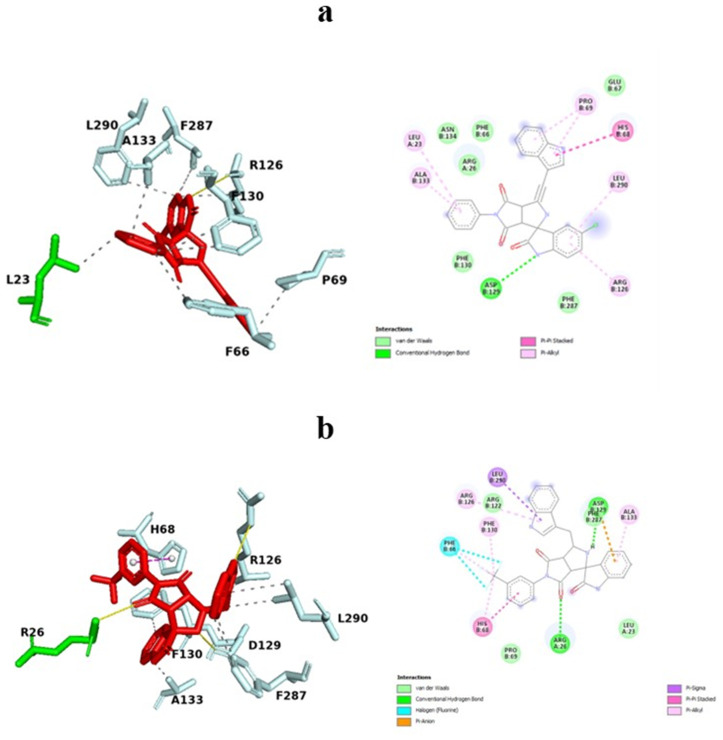
Visual representation of the binding interactions of compounds. (a) Z33 and (b) C2 within the cyclin D/CDK4 complex. The 3D binding interactions were generated using PyMOL, while 2D interactions were visualized with Discovery Studio Visualizer, detailing interactions with key residues in the active site.

Fig 5 displays the 3D and 2D representations of the interactions between C2 and 2W99, showing conventional hydrogen bonds, carbon-hydrogen bonds, halogen bonds, π-sigma interactions, alkyl interactions, π-alkyl interactions, π-sulfur interactions, and π-cation interactions. Similarly, Fig 5 also illustrates the 3D and 2D interactions between Z33 and 2W99.

### 3.5. Molecular dynamic simulation

Molecular dynamics simulations (MDS) are computer-based methods that provide detailed insights into the actual locations and movements of atoms and molecules. While molecular docking typically shows only one possible binding position for the ligand-protein complex, MDS allow for the observation of conformational changes and stability over time [34]. In docking, the inhibitor and protein interaction occur almost instantaneously, often leading to an unstable complex. MDS, however, can offer more detailed insights into which molecular interactions remain stable over time.

For this study, we used CHARMM-GUI to generate protein topology and ligand parameter files, and GROMACS-2019 was employed to perform the molecular dynamics simulations. To assess the system’s stability throughout the simulation, we monitored the root-mean-square deviation (RMSD) of the backbone structure relative to its initial position. Using a 100 ns MDS trajectory, we analyzed the overall conformational stability of the protein-ligand interactions. The RMSD, radius of gyration (RG), root mean square fluctuation (RMSF), and the number of hydrogen bonds were evaluated as functions of time.

The most stable interactions identified from the docking were further examined using the 100 ns MDS of the 2W99-C2 complex. The simulation showed that all candidate complexes displayed significant structural stability and compactness. After completing the simulation, we analyzed the RMSD, RG, RMSF, and hydrogen bond trajectories for both the complex and the reference protein.

**Fig 6:**
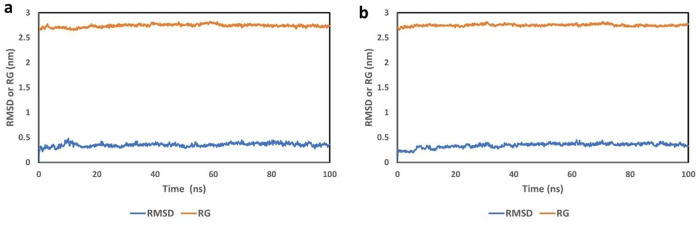
Comparative plots of root mean square deviation (RMSD) and radius of gyration (Rg) for (a) the 2W99 structure and (b) the 2W99-C2 complex, as assessed during the 100 ns molecular dynamics (MD) simulation.

Figs 6(a) and 6(b) present the RMSD and RG graphs for the 2W99 and 2W99-C2 complexes throughout the simulation. The RMSD values help assess whether the system’s parts remained balanced and if the protein conformation changed significantly during the simulation [35]. The RG plot provides further insight into the protein’s compactness during the simulation, which is important for understanding how the protein maintains its structure [36].

In Fig 6(a), the RMSD and RG values for the backbone of 2W99 (D-cyclin-CDK4) are shown as a reference. The RMSD fluctuated between 0 and 0.4 nm and remained stable from 10 to 100 ns with an average value of 0.34 nm, indicating good stability. The RG value showed an average of 2.73 nm after 30 ns, with low fluctuations, suggesting high compatibility and stability.

Fig 6(b) displays the RMSD and RG graphs for the 2W99-C2 complex. The RMSD remained between 0 and 0.4 nm, with an average of 0.34 nm, indicating stability throughout the simulation. The RG value for 2W99-C2 showed minimal fluctuation, high compatibility, and an average value of 2.75 nm over the 100 ns simulation, confirming that the complex remained stable and compact.

**Fig 7:**
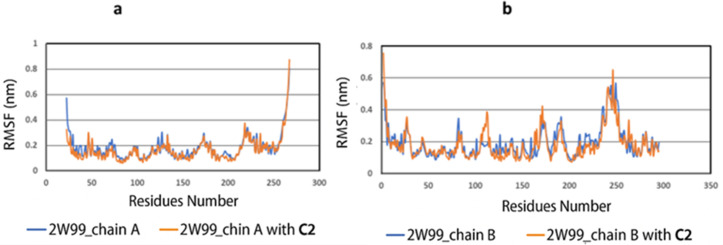
Root mean square fluctuation (RMSF) plots of chain A (cyclin D) and chain B (CDK4) in the 2W99 complex. Comparison between the unbound 2W99 structure and the 2W99-C2 complex for **(a)** chain A and **(b)** chain B, revealing conformational changes upon ligand binding.

A third analysis was conducted by generating RMSF graphs, which track local fluctuations in the protein’s amino acid residues during the simulation. Fig 7(a) shows the RMSF for chain A (D-cyclin), with an average of 0.177 nm, and the RMSF for chain A-C2, with an average of 0.163 nm. These results indicate minimal fluctuation and good stability compared to the reference. Similarly, Fig 7(b) shows the RMSF for chain B (CDK4) with an average of 0.194 nm, and for chain B-C2, with an average of 0.183 nm. In comparison to the backbone proteins, C2 with D-cyclin or CDK4 showed minimal RMSF values, indicating low divergence from the average position, low structural mobility, and high stability.

**Fig 8:**
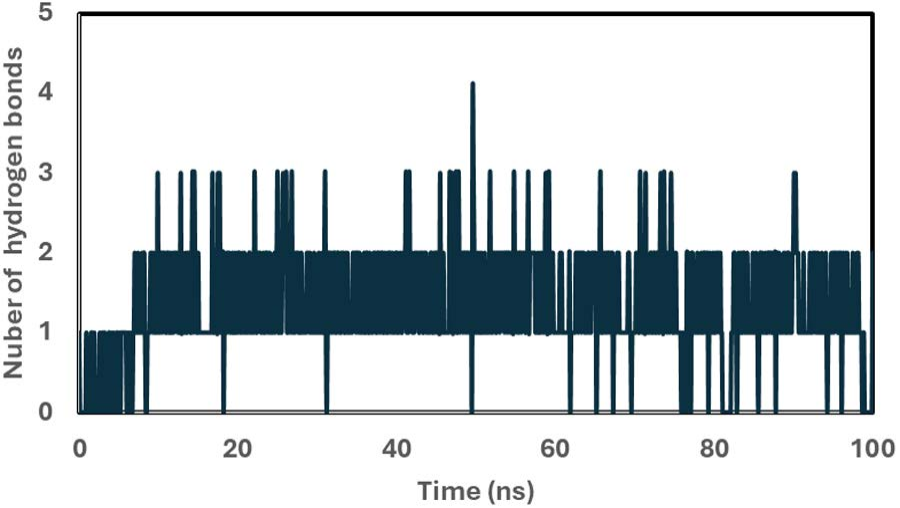
Time evolution of hydrogen bonds formed between the 2W99 complex and compound C2 during the 100 ns MDS, indicating the stability of crucial interactions over time.

Finally, hydrogen bond analysis was conducted to assess the stability of the 2W99-C2 complex. Hydrogen bonds play a crucial role in drug selectivity, metabolism, and adsorption [37]. Fig 8 shows the number of hydrogen bonds formed between C2 and 2W99 during the MDS. The 2W99-C2 complex showed an average of 1.34 hydrogen bonds, with values ranging from 0 to 4. This result suggests that the C2 inhibitor forms stable interactions with the protein, increasing the likelihood that C2 can effectively inhibit the D-cyclin-CDK4 complex and potentially be used in cancer treatment.

## 4. Conclusion

The cyclin D/CDK4 complex has become a promising target in cancer therapy, and our computational analysis highlights the potential of compound C2 as an inhibitor of this complex. Molecular docking studies demonstrated that C2 binds with high affinity to a key cavity within the D-cyclin-CDK4 complex, suggesting its potential effectiveness as an inhibitor. The ligand-protein interaction analysis revealed significant interactions, including hydrophobic contacts, π-cation interactions, hydrogen bonds, and halogen bonds, which further support its potential for experimental validation and in vitro studies. Molecular dynamics simulations confirmed the stability of the C2 binding, with favorable results in terms of radius of gyration (RG), root mean square deviation (RMSD), root mean square fluctuation (RMSF), and hydrogen bond formation. These findings suggest that compound C2 holds promise for cancer chemotherapy targeting the cyclin D/CDK4 complex. However, further experimental studies are needed to fully evaluate the biological and pharmacological properties of C2 in this context.

## References

[1] Dong P, Zhang C, Parker B-T, You L, Mathey-Prevot B. Cyclin D/CDK4/6 activity controls G1 length in mammalian cells. PLoS One. 2018;13(1):e0185637. doi: 10.1371/journal.pone.0185637 29309421 PMC5757913

[2] Malumbres M, Barbacid M. Cell cycle, CDKs and cancer: a changing paradigm. Nat Rev Cancer. 2009;9(3):153–66. doi: 10.1038/nrc2602 19238148

[3] van den Heuvel S, Dyson NJ. Conserved functions of the pRB and E2F families. Nat Rev Mol Cell Biol. 2008;9(9):713–24. doi: 10.1038/nrm2469 18719710

[4] Murray AW. Recycling the Cell Cycle. Cell. 2004;116(2):221–34. doi: 10.1016/s0092-8674(03)01080-8 14744433

[5] Narasimha AM, Kaulich M, Shapiro GS, Choi YJ, Sicinski P, Dowdy SF. Cyclin D activates the Rb tumor suppressor by mono-phosphorylation. eLife. 2014;3. doi: 10.7554/elife.02872 PMC4076869 24876129

[6] Huber K, Mestres-Arenas A, Fajas L, Leal-Esteban LC. The multifaceted role of cell cycle regulators in the coordination of growth and metabolism. FEBS J. 2021;288(12):3813–33. doi: 10.1111/febs.15586 33030287 PMC8359344

[7] Gao X, Leone GW, Wang H. Cyclin D-CDK4/6 functions in cancer. Adv Cancer Res. 2020;148:147–69. doi: 10.1016/bs.acr.2020.02.002 32723562

[8] Ameratunga M, Kipps E, Okines AFC, Lopez JS. To Cycle or Fight-CDK4/6 Inhibitors at the Crossroads of Anticancer Immunity. Clin Cancer Res. 2019;25(1):21–8. doi: 10.1158/1078-0432.CCR-18-1999 30224338

[9] O’Sullivan CC, Clarke R, Goetz MP, Robertson J. Cyclin-Dependent Kinase 4/6 Inhibitors for Treatment of Hormone Receptor-Positive, ERBB2-Negative Breast Cancer: A Review. JAMA Oncol. 2023;9(9):1273–82. doi: 10.1001/jamaoncol.2023.2000 37382948 PMC11385778

[10] VanArsdale T, Boshoff C, Arndt KT, Abraham RT. Molecular Pathways: Targeting the Cyclin D-CDK4/6 Axis for Cancer Treatment. Clin Cancer Res. 2015;21(13):2905–10. doi: 10.1158/1078-0432.CCR-14-0816 25941111

[11] Adon T, Shanmugarajan D, Kumar HY. CDK4/6 inhibitors: a brief overview and prospective research directions. RSC Adv. 2021;11(47):29227–46. doi: 10.1039/d1ra03820f 35479560 PMC9040853

[12] Dai M, Boudreault J, Wang N, Poulet S, Daliah G, Yan G, et al. Differential Regulation of Cancer Progression by CDK4/6 Plays a Central Role in DNA Replication and Repair Pathways. Cancer Res. 2021;81(5):1332–46. doi: 10.1158/0008-5472.CAN-20-2121 33372040

[13] Alinari L, Edwards RB, Prince CJ, Towns WH, Mani R, Lehman A, et al. Targeting the Cyclin D - CDK4/6 - Rb Axis in Mantle Cell Lymphoma with the Novel Translation Inhibitor Silvestrol,. Blood. 2011;118(21):3498–3498. doi: 10.1182/blood.v118.21.3498.3498

[14] Lu H, Chang DJ, Baratte B, Meijer L, Schulze-Gahmen U. Crystal structure of a human cyclin-dependent kinase 6 complex with a flavonol inhibitor, fisetin. J Med Chem. 2005;48(3):737–43. doi: 10.1021/jm049353p 15689157

[15] Nandi S, Dey R, Dey S, Samadder A, Saxena AK. Naturally Sourced CDK Inhibitors and Current Trends in Structure-Based Synthetic Anticancer Drug Design by Crystallography. Anticancer Agents Med Chem. 2022;22(3):485–98. doi: 10.2174/1871520621666210908101751 34503422

[16] Abdel-Magid AF. Potential of Cyclin-Dependent Kinase Inhibitors as Cancer Therapy. ACS Med Chem Lett. 2021;12(2):182–4. doi: 10.1021/acsmedchemlett.1c00017 33603963 PMC7883368

[17] Schrödinger LLC. The PyMOL molecular graphics system (Version 2.5). https://pymol.org

[18] Koes DR, Camacho CJ. ZINCPharmer: pharmacophore search of the ZINC database. Nucleic Acids Res. 2012;40(Web Server issue):W409-14. doi: 10.1093/nar/gks378 22553363 PMC3394271

[19] Hanwell MD, Curtis DE, Lonie DC, Vandermeersch T, Zurek E, Hutchison GR. Avogadro: an advanced semantic chemical editor, visualization, and analysis platform. J Cheminform. 2012;4(1):17. doi: 10.1186/1758-2946-4-17 22889332 PMC3542060

[20] Kundu D, Dubey VK. Potential alternatives to current cholinesterase inhibitors: an in silico drug repurposing approach. Drug Dev Ind Pharm. 2021;47(6):919–30. doi: 10.1080/03639045.2021.1952216 34219594

[21] Trott O, Olson AJ. AutoDock Vina: improving the speed and accuracy of docking with a new scoring function, efficient optimization, and multithreading. J Comput Chem. 2010;31(2):455–61. doi: 10.1002/jcc.21334 19499576 PMC3041641

[22] Morris GM, Huey R, Lindstrom W, Sanner MF, Belew RK, Goodsell DS, et al. AutoDock4 and AutoDockTools4: Automated docking with selective receptor flexibility. J Comput Chem. 2009;30(16):2785–91. doi: 10.1002/jcc.21256 19399780 PMC2760638

[23] Venkatesan SK, Shukla AK, Dubey VK. Molecular docking studies of selected tricyclic and quinone derivatives on trypanothione reductase of Leishmania infantum. J Comput Chem. 2010;31(13):2463–75. doi: 10.1002/jcc.21538 20340105

[24] Pande M, Kundu D, Srivastava R. Vitamin C and Vitamin D3 show strong binding with the amyloidogenic region of G555F mutant of Fibrinogen A alpha-chain associated with renal amyloidosis: proposed possible therapeutic intervention. Mol Divers. 2022;26(2):939–49. doi: 10.1007/s11030-021-10205-7 33710477

[25] Daina A, Michielin O, Zoete V. SwissADME: a free web tool to evaluate pharmacokinetics, drug-likeness and medicinal chemistry friendliness of small molecules. Sci Rep. 2017;7(1). doi: 10.1038/srep42717 PMC5335600 28256516

[26] Adasme-Carreño F, Muñoz-Gutierrez C, Caballero J, Alzate-Morales JH. Performance of the MM/GBSA scoring using a binding site hydrogen bond network-based frame selection: the protein kinase case. Phys Chem Chem Phys. 2014;16(27):14047–58. doi: 10.1039/c4cp01378f 24901037

[27] Pronk S, Páll S, Schulz R, Larsson P, Bjelkmar P, Apostolov R, et al. GROMACS 4.5: a high-throughput and highly parallel open source molecular simulation toolkit. Bioinformatics. 2013;29(7):845–54. doi: 10.1093/bioinformatics/btt055 23407358 PMC3605599

[28] Aziz NAAM, George RF, El-Adl K, Mahmoud WR. Design, synthesis, in silico docking, ADMET and anticancer evaluations of thiazolidine-2,4-diones bearing heterocyclic rings as dual VEGFR-2/EGFRT790M tyrosine kinase inhibitors. RSC Adv. 2022;12(20):12913–31. doi: 10.1039/d2ra01119k 35496328 PMC9045483

[29] Lipinski CA, Lombardo F, Dominy BW, Feeney PJ. Experimental and computational approaches to estimate solubility and permeability in drug discovery and development settings. Adv Drug Deliv Rev. 2001;46(1–3):3–26. doi: 10.1016/s0169-409x(00)00129-0 11259830

[30] Veber DF, Johnson SR, Cheng H-Y, Smith BR, Ward KW, Kopple KD. Molecular properties that influence the oral bioavailability of drug candidates. J Med Chem. 2002;45(12):2615–23. doi: 10.1021/jm020017n 12036371

[31] Egan WJ, Merz KM Jr, Baldwin JJ. Prediction of drug absorption using multivariate statistics. J Med Chem. 2000;43(21):3867–77. doi: 10.1021/jm000292e 11052792

[32] Muegge I, Heald SL, Brittelli D. Simple selection criteria for drug-like chemical matter. J Med Chem. 2001;44(12):1841–6. doi: 10.1021/jm015507e 11384230

[33] Ghose AK, Viswanadhan VN, Wendoloski JJ. A knowledge-based approach in designing combinatorial or medicinal chemistry libraries for drug discovery. 1. A qualitative and quantitative characterization of known drug databases. J Comb Chem. 1999;1(1):55–68. doi: 10.1021/cc9800071 10746014

[34] Fayyazi N, Fassihi A, Esmaeili S, Taheri S, Ghasemi JB, Saghaie L. Molecular dynamics simulation and 3D-pharmacophore analysis of new quinoline-based analogues with dual potential against EGFR and VEGFR-2. Int J Biol Macromol. 2020;142:94–113. doi: 10.1016/j.ijbiomac.2019.09.077 31521657

[35] Al-Zrkani MK, Abdulkareem RA, Al-Fahad D, Al Shouber M, Nasr AMS, Al-Khdhairawi A. Elucidating novel antibacterial compounds from the NPASS database against the FimH lectin domain for the treatment of urinary tract infections: an in-silico study. J Biomol Struct Dyn. 2023;41(9):3914–25. doi: 10.1080/07391102.2022.2059009 35403563

[36] Sargolzaei M. Effect of nelfinavir stereoisomers on coronavirus main protease: Molecular docking, molecular dynamics simulation and MM/GBSA study. J Mol Graph Model. 2021;103:107803. doi: 10.1016/j.jmgm.2020.107803 33333424 PMC7716089

[37] Rajagopal S, Vishveshwara S. Short hydrogen bonds in proteins. The FEBS Journal. 2005;272(8):1819–32. doi: 10.1111/j.1742-4658.2005.04604.x 15819878

